# Impact of mental health, job insecurity, and COVID-19 symptoms on protective behavior changes among White, Black, and other minorities in the US

**DOI:** 10.3389/fpsyg.2022.1040413

**Published:** 2022-11-21

**Authors:** Yingying Sun, Ping Wang, Jun Tang

**Affiliations:** ^1^School of Public Administration and Policy, Renmin University of China, Beijing, China; ^2^College of Business, James Madison University, Harrisonburg, VA, United States

**Keywords:** protective behavior, race, mental health, job insecurity, COVID-19 symptoms

## Abstract

**Introduction:**

Job insecurity such as loss of jobs or reduced wages has become a serious social problem in the US since COVID-19 started. Combined with psychological distress and experience of COVID-19 symptoms, the changes of people’s protective behaviors vary across states in the US.

**Methods:**

This research investigated racial differences in the COVID-19 related factors among White, Black, and other minorities in the US, and examined how mental health mediated the impact of job insecurity on protective behaviors, and how the COVID-19 symptoms moderated the mediation effect of mental health. The 731 valid responses in a cross-sectional survey from May 23 to 27, 2020, in the US were analyzed with independent sample t-tests, Pearson’s chi-square tests, and path analysis.

**Results:**

The findings showed that there were significant differences in job insecurity and Nonpharmaceutical Interventions (NPIs) practice among White, Black, and other minorities. Job insecurity was significantly negatively associated with NPIs practice and was significantly positively associated with mental health. Mental health significantly partially mediated the effect of job insecurity on NPIs practice, in that job insecurity is a better predictor of NPIs practice for individuals with worse mental health than that for individuals with better mental health. Experience of COVID-19 symptoms moderates the mediation effect of mental health on the relationship between job insecurity and NPIs practice, in that mental health is a better predictor of NPIs practice for individuals with a higher experience of COVID-19 symptoms than for individuals with a lower experience of COVID-19 symptoms.

**Discussion:**

The findings in this study shed lights on psychological and behavioral studies of people’s behavior changes during a pandemic. The study indicates the importance of treating mental health to promote protective behaviors during a pandemic, as well as advocating for employees by identifying the needs for those whose jobs were negatively impacted the most.

## Introduction

The COVID-19 pandemic has had a huge impact on people’s lives. As of early September 2022, there were more than 6.484 million deaths, and at least 603.7 million confirmed cases of COVID-19 infections in the world (WHO, 2022). A wide range of protective measures including self-isolation, mass quarantine, travel restriction, community lockdown, and establishment of isolation units have been implemented to mitigate the COVID-19 risk across the globe during various stages of the pandemic (Bouey, 2020; Aykanian, 2022). These protective measures, the highly contagious, rapid ascent, and being fast mutation and more transmissible nature of the virus required changes in people’s behavior (Lüdecke and von dem Knesebeck, 2020; Lu and Lin, 2021). These behavior changes included wearing face masks in public, distancing socially, staying at home, and practicing hand hygiene (Ammar et al., 2020; Adiyoso and Wilopo, 2021). Meanwhile, recent evidence reveals that those containment measures have exerted huge negative impacts on economic activities and employment market (Ganson et al., 2021; Lin et al., 2021). Such worsened economic situation may have had adverse psychological influences on people’s mental health (Jacobson et al., 2020; Fu et al., 2021), such as stress, depression, and anxiety (Kantor and Kantor, 2020; Liu et al., 2022). Nevertheless, impacts of COVID-19 might not be equally experienced by the global population, as those containment measures were implemented differently across countries (Zhang et al., 2020, 2021).

As the US has reported the largest number of COVID-19 death and confirmed cases, it is important to further explore the impact of COVID-19 on people’s protective behavior changes and study relevant factors in the US. The current study aims to explore possible influencing factors on the changes of protective behaviors during COVID-19. By paying special attention to the official control measures of COVID-19 responses, this study reviews the negative effects on public protective behavior changes caused by job insecurity, mental health, and experience of COVID-19 symptoms. Further, the associations among those effects and people’s protective behavior changes are examined.

### NPIs and racial disparity

Protection Motivation Theory (PMT) explains how people choose to protect themselves to reduce environmental risks (Maddux and Rogers, 1983; Norman et al., 2005). PMT argues that people take adaptive actions on the base of estimating the degree of threat and assessing their abilities to perform protective behaviors (Al-Rasheed, 2020). To prevent being infected and slow down transmission of the virus, World Health Organization (WHO) and the US Centers for Disease Control and Prevention (CDC) have recommended protective measures of NPIs such as keeping a distance of at least 1 m from others, staying at home or self-isolating at home, regularly washing hands with soap and water, and wearing face masks when going outside (CDC, 2020; WHO, 2020). As the federal and state governments did not impose lockdown policies, effective control over the spread of the virus and reducing the impacts of the pandemic will be dependent on public behavior changes (Wise et al., 2020; Sun et al., 2021). Despite the importance of NPIs practice in mitigating the COVID-19 risk, there still is a quite high proportion of people who refused wearing facemasks and failed keeping social distances (Sun et al., 2021). For example, a longitudinal study in the US which recruited 1,591 subjects found that people engaged in protective behaviors such as maintaining social distances and practicing hand hygiene more than usual, while a subgroup of individuals did not engage in these behaviors (Wise et al., 2020). PMT states that people may be more likely to comply with health-related measures if they had been affected by the outbreak, and if they perceive that the illness has severe consequences (Milne et al., 2000; Rubin et al., 2009). It is, therefore, critical to explore factors, such as mental health, job insecurity and protective behavior changes, to mitigate the risk of spreading COVID-19 virus.

Communities of color in the US have been disproportionately affected by the pandemic, such that Latins and Hispanics were 10 times more likely to experience depression than Whites (Saltzman et al., 2021). The National Center for Health Statistics (NCHS, 2022) revealed that, among the COVID-19 fatality, 21.5% were Black and 27.7% were Hispanics. This may be due to the enduring structural inequalities (i.e., lack of access to medical and financial resources) for the Black and Hispanic populations which led to the increased risk of exposure to COVID-19 (Andrasfay and Goldman, 2021). The Chicago public record of COVID-19 deaths shows that, Black residents (31% of the population) accounted for 42% of the deaths, and higher mortality was seen in neighborhoods with heightened barriers to social distancing and low health insurance coverage (Scannell Bryan et al., 2021). A recent study on Americans aged 51 and older showed that, the associations of agreeableness with handwashing and physical distancing were weaker for Hispanic older adults than their non-Hispanic White counterparts (Choi et al., 2022). While those public reports have highlighted the existence of racial disparities during COVID-19, it is important to consider how NPIs were practiced among different racial groups (Smith et al., 2022). The current study investigates the differences of NPIs practice, job insecurity, mental health, and experience of COVID-19 symptoms among Whites, Blacks, and other minorities in the US. Therefore, this study aims to answer the following research question:

RQ: Are there differences in the degree of NPIs practice, job insecurity, mental health, and experience of COVID-19 symptoms among White, Black, and other minorities?

### Job insecurity during the height of the COVID-19 pandemic

During the height of the pandemic in 2020, containment measures varied across states in the US, which included quarantine, temporary and indefinite closure of non-essential businesses, and promotion of protective behaviors such as practicing hand hygiene and wearing face masks (O’Conor et al., 2020). During this period, job insecurity and the subsequent loss of health insurance were of concerns among those working on jobs with minimum wage without options for remote work access (Wilson et al., 2020; Lin et al., 2021). Reports from the National Bureau of Economic Research showed that many businesses closed temporarily or permanently between February and May 2020, resulting in the layoff or furlough of 10.1% of US workers (Brynjolfsson et al., 2020). As essential businesses, such as grocery stores, were allowed to stay open during the pandemic, wage-earning employees were forced to work throughout the pandemic, as they risked losing hourly wages, or becoming unemployed, if they did not go to work as scheduled (Ganson et al., 2021; Aykanian, 2022). Additionally, many other wage-earning employees (e.g., food servers) lost their jobs as their places of employment (e.g., restaurants) were forced to close indefinitely during the pandemic (Saenz and Sparks, 2020). Therefore, people who had to work through the pandemic put themselves at a greater risk of contracting COVID-19 and spreading the virus to their household members (Aykanian, 2022). Additionally, those who lost their jobs without a steady income worried about paying bills, such as rent, food, and other household expenses during the pandemic (Wilson et al., 2020). Job insecurity and financial concern during the COVID-19 pandemic could result in resistance of practicing NPIs (Gemelas et al., 2021).

### Mental health problems due to COVID-19

The COVID-19 pandemic and worsened economic activities have been associated with mental health disorders (e.g., depression, isolation) (McCray and Rosenberg, 2021; Veldhuis et al., 2021). Together with anxiety about catching the virus, financial concerns, and loss of family members, the pandemic has had a dramatic effect on the mental health of millions, including ethnic minorities, medical staffs, and the public (Diamond and Byrd, 2020). Doctors who were working during the pandemic have a high prevalence of depression, stress, and anxiety because of long duty hours and high-risk duties such as fever clinic and isolation ward (Chatterjee et al., 2020). As the pandemic continues impairing daily livings, Black, Hispanics, Asian, and bi-multiracial groups appeared to be at higher risk of distress like isolation, and hopelessness than White (Veldhuis et al., 2021). As the first COVID-19 case was reported in China, Asian populations have experienced discrimination in the US, which has been associated with anxiety and other mental health conditions (Liu et al., 2020). To understand the mental health status of ethnic groups is a priority to enact supportive social policies, as supportive policies weaken the association between household income shocks and mental health (Donnelly and Farina, 2021). The current study examines the mental health differences among White, Black, and other minorities in the US.

As described above, the enactment of containment measures to protect the public from COVID-19 negatively affect mental health (Jacobson et al., 2020; Kecojevic et al., 2020). With poorer mental health, people were more likely to be drinking alcohol more than they used to (Niedzwiedz et al., 2021). Examined behavior changes of 13,829 respondents, an Australian study reported that one in five adults drank more alcohol since the pandemic began than they used to if they had more severe symptoms of depression or anxiety (Tran et al., 2020). These studies focused on the impacts of mental health on people’s behavior changes in regard to alcohol, smoking, or drug use. Meanwhile, many studies have examined the impact factors on mental health, such as demographic factors, self-efficacy, and knowledge of COVID-19 (Wang et al., 2021; Yıldırım and Güler, 2022). Quite a few studies have investigated the impact factors on NPIs practice, such as risk perception, policy measures, experience of virus outbreak, and information source (Ammar et al., 2020; Sun et al., 2021). Only a few studies have examined the mental health impact on NPIs practice (Mata et al., 2021). This study filled the void to examine the association of mental health and NPIs practice and study the mediation effect of mental health between job insecurity and NPIs practice.

At the start of the pandemic, the unemployment rate reached a record-high of 14.7%, and approximately one in five workers claimed unemployment insurance as of July 2020 in the US (US Bureau of Labor Statistics, 2020). The higher dramatic declines in the number of employments were seen in the Black, Asian American, and Hispanics than White (Gemelas et al., 2021). Job insecurity activated a series of adversities such as financial strain, lowered self-esteem, and family disruption that undermine mental health (McDowell et al., 2021). Even the employees’ perceived COVID-19 disruption was positively related to their attitudes towards job insecurity, which in turn was positively related to their emotional exhaustion (Lin et al., 2021). A US study of 2,301 participants further confirmed that, compared to people whose employment remained unchanged, people who switched to work from home style did not differ in any measures of mental health, but people who had lost their jobs reported higher symptoms of depression and stress (McDowell et al., 2021).

### Experience of COVID-19 symptoms

When the fatality rate of the virus was high during the early stages of the pandemic, people’s mental health was likely to be influenced by their experience of COVID-19 symptoms (Wilson et al., 2020). COVID-19 is a respiratory disease with common symptoms, including fever, cough, and difficulty of breathing (Guan et al., 2020; Huang et al., 2020), which are similar to other common afflictions such as influenza, head colds and allergies. Based on the theory of PMT, people’s personal beliefs and values determine how they view and evaluate potential health risks, thus affecting their personal protective behavior (Norman et al., 2005). Facing a new health risk such as COVID-19 symptoms, people’s on-situ experiences would play a guiding role with increased fluctuations in their emotional changes, ups and downs of their mental health and lead to protective behavior changes. Therefore, the interactions of experience of COVID-19 symptoms and mental health were likely to impact protective behavior changes such as NPIs practice.

To protect the public against the virus, NPIs, such as facemasks wearing, body temperature monitoring, and social distancing were promoted (Hornik et al., 2021; Yang et al., 2021). Evidence shows that daily contacts can be reduced seven to eight folds when the social distancing orders were enacted (Zhang et al., 2020). Though, the observation and experience of virus symptoms could encourage people to engage in protective behaviors such as practicing NPIs, people would only be willing to practice certain types of NPIs (Adiyoso and Wilopo., 2021). The US residents’ experience of COVID-19 symptoms might vary greatly, as significantly higher rates of COVID-19 diagnosis were in Black communities (Millett et al., 2020). A campus study in the US during the 2009 pandemic influenza A (H1N1) reported that, 3,924 (65%) of 6,049 student respondents and 1,057 (74%) of 1,401 faculty respondents increased their use of NPIs such as practicing hand hygiene, but for people who had infected with the virus, only no more than 10% individuals reported staying home while ill (Mitchell et al., 2011). People who believed that they had infected with COVID-19 were less likely to report adhering to recommended NPIs (Smith et al., 2022). This is important as an individual’s experience of COVID-19 symptoms may impact their practice of NPIs such as facemask wearing and social distancing. Based on the above findings in the literature, the following hypotheses are, therefore, proposed accordingly:

*Hypothesis 1*: There is a significant negative association between job insecurity and NPIs practice.

*Hypothesis 2*: There is a significant positive association between job insecurity and mental health.

*Hypothesis 3*: Mental health significantly mediated the impact of job insecurity on NPIs practice.

*Hypothesis 4*: Experience of COVID-19 symptoms significantly moderated the mediation effect of mental health between job insecurity and NPIs practice.

To fill the void of research gap in examining possible associations among protective behavior changes, job insecurity, and mental health, the current study contributes to the psychological and behavior changes literature in several ways: 1) the specific examination of the association between job insecurity and mental health could provide evidence about the importance of maintaining economic activities during a pandemic; 2) the mental health’s mediating role between job insecurity and protective behavior changes could further reveal serious consequences of prolonged mental health and the need for improved mental health care during a pandemic; 3) the study of how COVID-19 symptoms and mental health interactively impact the relationship between job insecurity and protective behavior changes could present additional evidence about the need for continued effort to reduce the risk of being infected; 4) the findings of this study could have theoretical and practical implications for future pandemic response to consider more assertive strategies to mitigate the infection risk, proactively deal with mental health, and reduce the negative impact due to slowed down of economic activities.

## Materials and methods

### Data collection

A cross-sectional survey was administered online *via* Qualtrics XM platform from May 23 to 27, 2020. Respondents who were at least 18 years of age, lived in the US, and confirmed the informed consent before accessing the survey. The survey was made up of five sections related to the COVID-19 pandemic, including NPIs practice, job insecurity, mental health, experience of COVID-19 symptoms and demographic data (e.g., age, gender, and education). Qualtrics generated a convenient sample that was roughly representative of the US adult population based on age distribution. The research protocol was approved by the US university’s Institutional Review Board. Among a total of 921 respondents who started the survey, 731 were deemed valid, giving a response rate of 79.4%.

### Measurements

#### NPIs practice

Built on recommended NPIs items (CDC, 2020), the question required respondents to rank the degree to which they have practiced NPIs on a 7-point Likert scale (1 = not at all, 7 = very frequently). Items of NPIs included (1) reduce family/friend gatherings, (2) reduce the use of public transportation, (3) social distancing by staying at least 1 m away, (4) social distancing by avoiding gathering in groups, and (5) wear facemasks. The factor loadings of these items showed that, only the former four items had robust loadings. This might be due to the confusions on the use of facemasks during the COVID-19 pandemic in the US (Hornik et al., 2021; Yang et al., 2021). A higher level of responses indicated more practice of NPIs.

#### Job insecurity

Job insecurity was measured through four questions: “Have you been affected by the COVID-19 pandemic in the form of 1) employer bankruptcy, 2) loss of clients or customers, 3) wage reduction, 4) lay off, or no contract renewal after its expiration?” Answers to each of these questions were measured using a 7-point Likert scale (1 = not at all, 7 = very negatively impacted). A higher level of response indicated more severe insecurity of employment.

#### Mental health

The survey used the K6 questionnaire of psychological distress to measure mental health disorders. The K6 questionnaire was used in the US National Health Interview Survey and the US National Household Survey of Drug Abuse (Kessler et al., 2002). The K6 asks “How often did: (1) you feel so depressed that nothing could cheer you up, (2) you feel hopeless, (3) you feel restless or fidgety, (4) you feel that everything was an effort, (5) you feel worthless, (6) you feel nervous.” Each response was measured using a 7-point Likert scale (1 = not at all, 7 = all the time). A higher level of response indicated worse mental health. Results of the factor loadings showed that, the former two items did not have higher enough factor loadings on measuring mental health. The current study used the later four items in the analysis.

#### Experience of COVID-19 symptoms

Built on established COVID-19 symptoms guidelines (Guan et al., 2020), the question was created as, “To what degree have you or the people you live with had any of the following symptoms during the pandemic: fever, respiratory tract infection, cough, shortness of breath or difficulty of breathing, or new loss of taste or smell?” Answers to the question were measured using a 7-point Likert scale (1 = no symptoms, 7 = severe symptoms). A higher level of response indicated more severe experience of COVID-19 symptoms.

#### Demographic factors

Race was measured by asking participants to select one from five options of White, Black, Asian, Hispanic, and other race. Due to the smaller portion of responses in the sample from Asians, Hispanics and other race, these three races were combined into “other races.” Gender was measured by male and female. Regarding age, participants were asked to select one from five options of 18–30 years, 31–40 years, 41–50 years, and at least 51 years. Regarding marital status, participants were asked to select one from five options, and the marital status variable was recoded into 1 = married and 2 = unmarried (including single, divorced, widowed, and others). Regarding education, participants were asked to select one from five options, and the education variable was recoded into 1 = no more than high school (including no more than middle school, high school), 2 = two-year college degree, and 3 = at least undergraduate degree (including undergraduate degree, at least graduate degree). Regarding main occupation, participants were asked to select one from seven options, and the occupation variable was recoded into 1 = student, 2 = employed (including farming, self-employed, regular jobs, odd jobs), 3 = unemployed, 4 = retired. The presence of chronic disease (e.g., hypertension and diabetes) increases the risk of severe COVID-19, which means a greater risk of hospitalization and death and may have effect on the adoption of NPIs (Cai et al., 2021). To determine the number of household members with underlying chronic disease, the question was asked as: “Including yourself, how many people in your household have a chronic disease that needs ongoing treatment and medication, such as diabetes, cancer, etc.?” The possible answer options were given as 0 = none, and 1 = one or more people. As age brings a higher risk of chronic disease and is associated with severe COVID-19 (Bhargava et al., 2021), households with older adults >65 years might have developed mental health disorders during the pandemic. To determine the number of household members >65 years old, the question was asked as “Including yourself, how many people older than 65 are in your household?” The possible answer options were given as 0 = none, and 1 = one or more people.

### Data analysis

Descriptive analyses were performed using frequency, percentage, mean, and standard deviation (SD). Independent sample t-tests and Pearson’s chi-square tests were conducted to examine differences of NPIs practice and the predicted factors among White, Black, and other minorities with SPSS 27 (IBM, Armonk). The level of statistical significance was set as *p* < 0.05. Path analysis was performed to examine the impact of job insecurity on NPIs practice. The mediating role of mental health between job insecurity and NPIs practice was examined and conducted with LISREL 9.32.^1^ The moderating role of experience of COVID-19 symptoms for the mediation effect of mental health between job insecurity and NPIs practice was examined and conducted with SmartPLS.^2^

## Results

### Demographics of respondents

As shown in Table 1, the majority of respondents were White (*n* = 493, 67.4%), female (*n* = 443, 60.6%) and unmarried (*n* = 432, 59.1%). Half of the respondents were employed (*n* = 380, 52.0%), which indicated that the job insecurity situation may be serious. There were 298 (40.8%) respondents reported having household members with chronic disease and 243 (33.2%) reported having household members over 65 years of age. White respondents reported the largest proportion from 31 to 40 years old (*n* = 162, 32.9%), while for Blacks and other minorities, the largest proportions of respondents were ranged from 18 to 30 years (*n* = 63, 56.8% for Black and *n* = 80, 63.0% for other minorities). These results revealed that there were more young respondents of Blacks and other minorities than those of Whites. Most Black respondents had no more than high school education (*n* = 54, 48.6%), while most White respondents (*n* = 202, 41.0%) and other minorities (*n* = 50, 39.4%) had at least an undergraduate education. These results indicated that Black respondents had lower education than the other two groups.

**Table 1:** Descriptive analysis results (*N* = 731).

	Total		White		Black		Other minority	
	*n* (%)	Mean (SD)	*n* (%)	Mean (SD)	*n* (%)	Mean (SD)	*n* (%)	Mean (SD)
**Race**								
White	493 (67.4)		493 (100)		0		0	
Black	111 (15.2)		0		111 (100)		0	
Other minorities	127 (17.4)		0		0		127 (100)	
**Gender**								
Male	288 (39.4)		197 (40.0)		475 (42.3)		44 (34.6)	
Female	443 (60.6)		296 (60.0)		64 (57.7)		83 (65.4)	
**Marital status**								
Married	299 (40.9)		238 (48.3)		29 (26.1)		32 (25.2)	
Unmarried	432 (59.1)		255 (51.7)		82 (73.9)		95 (74.8)	
**Age (year)**								
18–30	266 (36.4)		123 (24.9)		63 (56.8)		80 (63.0)	
31–40	231 (31.6)		162 (32.9)		32 (28.8)		37 (29.1)	
41–50	86 (11.8)		71 (14.4)		10 (9.0)		5 (3.9)	
51+	148 (20.2)		137 (27.8)		6 (5.4)		5 (3.9)	
**Occupation**								
Student	101 (13.8)		44 (8.9)		23 (20.7)		34 (26.8)	
Employed	380 (52.0)		246 (49.9)		61 (55.0)		73 (57.5)	
Unemployed	118 (16.1)		85 (17.2)		18 (16.2)		15 (11.8)	
Retired	132 (18.1)		118 (23.9)		9 (8.1)		5 (3.9)	
**Education**								
No more than high school	273 (37.3)		173 (35.1)		54 (48.6)		46 (36.2)	
Two-year college	177 (24.2)		118 (23.9)		28 (25.2)		31 (24.4)	
At least undergraduate	281 (38.4)		202 (41.0)		29 (26.1)		50 (39.4)	
**Chronic disease**								
Yes	298 (40.8)		194 (39.4)		51 (45.9)		53 (41.7)	
No	433 (59.2)		299 (60.6)		60 (54.1)		74 (58.3)	
**Household members 65+**								
Yes	243 (33.2)		170 (34.5)		35 (31.5)		38 (29.9)	
No	488 (66.8)		323 (65.5)		76 (68.5)		89 (70.1)	
Mental health (4–28)		16.2 (5.9)		16.2 (6.1)		15.9 (6.0)		16.6 (5.4)
Job insecurity (4–28)		11.80 (7.4)		11.0 (7.5)		13.4 (7.0)		13.6 (7.1)
COVID-19 symptoms (1–7)		5.32 (1.98)		5.5 (2.0)		4.8 (2.0)		5.1 (1.9)
NPIs practice		33.7 (8.1)		34.4 (7.6)		31.8 (9.1)		32.7 (8.6)

### Racial differences in related factors

Table 1 has the means and standard deviations of mental health, job insecurity, COVID-19 symptoms, and NPIs practice. Table 2 shows the results of independent sample t-tests. Independent sample t-tests revealed that, NPIs practice (*p* = 0.006), job insecurity (*p* = 0.002), and experience of COVID-19 symptoms (*p* = 0.005) were significantly different between White and Black respondents. Similarly, NPIs practice (*p* = 0.043) and job insecurity (*p* < 0.001) were significantly different between White and other minorities. There exists not enough evidence to support any significant difference in mental health between White and Black respondents, and between White and other minorities. The tests of latent mean differences among these three racial groups confirmed above findings. These findings directly answered the research question, that there were racial differences in NPIs practice, job insecurity, and experience of COVID-19 symptoms. As shown in Table 2, the results of Pearson’s Chi-square tests indicated that the demographic factors, age, occupation, and marital status were significantly different between White and Black respondents, and between White and other minorities. Education (*p* = 0.008) was significantly different between White and Black respondents.

**Table 2:** Results of independent samples t-tests and Pearson’s chi-square tests.

	White (*n* = 493) vs. Black (*n* = 111)			White (*n* = 493) vs. Other minority (*n* = 127)		
	*t*-value	chi-square	*p*-value	*t*-value	chi-square	*p*-value
COVID-19 symptoms	2.83**		0.005	1.999		0.058
Job insecurity	−3.057**		0.002	−3.604***		<0.001
Mental health	0.450		0.653	−0.676		0.499
NPIs practice	2.787**		0.006	2.041*		0.043
Age	7.668***		<0.001	11.426***		<0.001
Chronic disease		1.634	0.201		0.239	0.625
Household members 65+		0.352	0.553		0.943	0.332
Occupation		22.670**	<0.001		48.898**	<0.001
Marital status		48.112***	<0.001		48.613***	<0.001
Gender		0.214	0.644		1.200	0.273
Education		9.716**	0.008		0.110	0.947

*** *p* < 0.001;

** *p* < 0.01;

* *p* < 0.05.

### Path analysis of the moderated mediation effect

To further study the impact of job insecurity on mental health, and the mediation effect of mental health and the moderation effect of COVID-19 symptoms on NPIs practice, a path analysis was conducted. The assessment of the measurement model fit was carried out by LISREL and SmartPLS. As mentioned above, even though significant latent mean differences were observed between White, Black, and other minorities, consistent permutation multigroup analysis with 5,000 bootstrapping runs *via* SmartPLS indicated there were not any differences in all path coefficients, except the one between Black and other minorities (*p* = 0.033). Therefore, the SmartPLS was used to conduct a 5,000-run of bootstrapping procedure to obtain the measures of the path coefficients, and confidence intervals for the structural model of data with 731 responses.

### Assessment of measurement model

Both univariate and multivariate normality were evaluated before assessing the measurement model. A data set is deemed normally distributed if the measures of its univariate and multivariate normality are within the allowable ranges (Mardia, 1974; Finney and DiStefano, 2013; Cain et al., 2017). The univariate normality is ensured if the univariate skewness has its absolute values not larger than 2 and the univariate kurtosis has its absolute values not larger than 7 (Finney and DiStefano, 2013). Table 3 shows that the values of the univariate skewness (ranged from −1.291 to 0.723) and the univariate kurtosis (ranged from −1.291 to 0.827) were within their corresponding acceptable ranges. Therefore, the evidence indicated that the dataset is not severely univariate non-normally distributed. The multivariate normality as measured by the Mardia’s normalized multivariate kurtosis coefficient with a value of 44, however, is far larger than the recommended cutoff value of 3 and shows enough evidence to believe the existence of the severe multivariate non-normality of the dataset (Mardia, 1974; Cain et al., 2017). The reported model fit indices in this study were based on the robust ML estimate with the Satorra-Bentler adjusted χ^2^ as suggested by Finney and DiStefano (2013). The LISREL 9.32 was used to estimate the measurement model here. The covariance matrix of all survey items was shown in Appendix A.

**Table 3:** Measurement items.

Construct	Indicators	Mean	Standard deviation	Skewness	Kurtosis	VIF	Standardized loading
Job insecurity	Have you been affected by any of the following during the COVID-19 pandemic? 1 (Not negatively impacted) to 7 (Very negatively impacted)						
	Job1: Employer bankruptcy	2.767	2.033	0.688	−0.892	4.729	0.9250
	Job2: Loss of clients or customers	2.661	1.920	0.723	−0.784	4.392	0.9105
	Job3: Wage reduction	3.276	2.190	0.359	−1.291	3.033	0.7700
	Job4: Lay off, or no contract renewal after its expiration	3.098	2.188	0.504	−1.187	2.940	0.7653
Mental health	How often do you feel the following symptoms? 1 (Not at all) to 7 (All the time)						
	MH1: You feel restless or fidgety	4.282	1.744	−0.334	−0.653	1.795	0.7003
	MH2: You feel that everything was an effort	3.999	1.890	−0.083	−1.033	1.902	0.7226
	MH3: You feel worthless	3.944	1.781	−0.044	−0.856	2.298	0.8090
	MH4: You feel nervous	3.990	1.776	−0.093	−0.855	2.369	0.8291
NPIs	To what degree have you practiced the following behaviors? 1 (Very little) to 7 (Very high)						
	NPI1: Reduced family or friends’ gatherings	5.499	1.692	−0.974	−0.004	2.239	0.7875
	NPI2: Reduce the use of public transportation	5.822	1.636	−1.291	0.827	1.936	0.7319
	NPI3: Keep social distancing - stay out of crowded places, and / or stay at least 6 feet from other people	5.576	1.731	−1.172	0.455	2.976	0.8860
	NPI4: Keep social distancing - do not gather in groups	5.692	1.713	−1.224	0.501	2.547	0.8330

The common method bias (CMB) was presented due to the measurement error when informants responded to survey questions about their attitudes, beliefs, and perceptions and at the same time evaluated their owns or their institutions’ performance (Podsakoff et al., 2003; Kim and Daniel, 2020). To assess the CMB based on Harman’s single-factor test (Podsakoff et al., 2003), an exploratory factor analysis was carried out. The results indicated that three distinct factors extracted with eigenvalues above 1 explained 74.50% of the total variance with the first factor explained 34.64% of the total variance, which was less than the majority of 50% of the total variance. A confirmative factor analysis (CFA) for a single-factor model was evaluated as well (Sanchez and Brock, 1996). The model fit indices of the CFA showed clearly a poor model fit with χ^2^_(119)_ = 3,215, RMSEA = 0.283, TLI = 0.183, CFI = 0.332, and SRMR = 0.2816, which further indicated that the single-factor model could not be used to explain the three constructs in the study. Therefore, there was not a serious issue of the CMB in this study.

The McDonald Omega (ω) was used to measure the construct internal consistency reliability (McDonald, 1999). The reliability procedure in JASP^3^ was used to calculate the values of the McDonald Omega (ω) and the corresponding 95% confidence intervals as shown in Table 4. The data showed acceptable internal consistency reliability with the values of the McDonald Omega (ω) ranged from 0.832 to 0.867 for mental health, from 0.893 to 0.916 for job insecurity, and from 0.873 to 0.900 for NPIs practice, which were all above the threshold of 0.7 (Hair et al., 2014). The convergent validity was assessed to be adequate by the average variance extracted (AVE) and the composite reliability (CR) (Fornell and Larcker, 1981). As shown in Table 4, the constructs’ AVE values of 0.596 for mental health, 0.635 for NPIs, and 0.717 for job insecurity were all above the acceptable level of 0.50; the CR values of 0.854 for mental health, 0.910 for job insecurity, and 0.940 for NPIs were all above the acceptable level of 0.70.

**Table 4:** Construct reliability and validity.

	Construct reliability and validity			Discriminant validity (Fornell - Larcker criterion)		
	McDonald’s ω and 95% CI	Composite reliability	Average variance extracted (AVE)	Job insecurity	Mental health	NPIs
Job Insecurity	0.904 (0.893, 0.916)	0.909	0.716	**0.846**		
Mental Health	0.850 (0.832, 0.867)	0.851	0.589	0.391	**0.767**	
NPIs	0.886 (0.873, 0.900)	0.885	0.659	−0.190	0.087	**0.812**

The diagonal values in **bold** fonts are the square roots of AVEs by Fornell - Larcker criterion, the off-diagonal values are the latent variables’ correlations.

The discriminant validity was assessed by higher amount of the square root values of AVEs over the corresponding constructs’ correlations (Fornell and Larcker, 1981) and the Heterotrait-Monotrait ratio of the cross constructs’ correlations (HTMT) less than the threshold value of 0.85 (Henseler et al., 2015). Table 4 showed that all square root values of AVEs from 0.767 for mental health to 0.846 for job insecurity were much higher than the correlations of corresponding constructs. Table 5 showed that the HTMT values ranged from 0.092 to 0.549 were smaller than the threshold value of 0.85 as suggested by Kline (2011). These results indicated that the three constructs had adequate discriminant validity.

**Table 5:** Discriminant validity with Heterotrait–Monotrait ratio (HTMT).

	Job insecurity	Mental health	NPIs	COVID-19 symptoms
Job insecurity				
Mental health	0.549			
NPIs	0.128	0.092		
COVID-19 symptoms	0.532	0.353	0.106	
Symptoms × Mental health	0.356	0.043	0.131	0.259

The model fit indices for the measurement model in this study included absolute fit index RMSEA = 0.081 [a cutoff value was suggested as 0.06 (Hu and Bentler, 1999), and 0.05–0.08 by Browne and Cudeck, 1993]; the incremental fit index TLI = 0.932 and the comparative fit index CFI = 0.948 (a cutoff value of 0.95 was suggested for both TLI and CFI by Hu and Bentler (1999)); and another absolute fit index SRMR = 0.048 (a cutoff value of 0.08 was suggested by Hu and Bentler (1999)). These model fit indices indicated the acceptable measurement model fit of the response data. The power of test was calculated as 1.000 with RMSEA = 0.081, significance level α = 0.05, sample size *n* = 731, and degree of freedom =51; the minimum sample size with a power of 0.80 is 200; and indicated a higher enough probability for a false null hypothesis to be rejected and to ensure the adequate sample size was used (MacCallum et al., 1996).

### Assessment of influencing factors on NPIs practice

**Figure 1:**
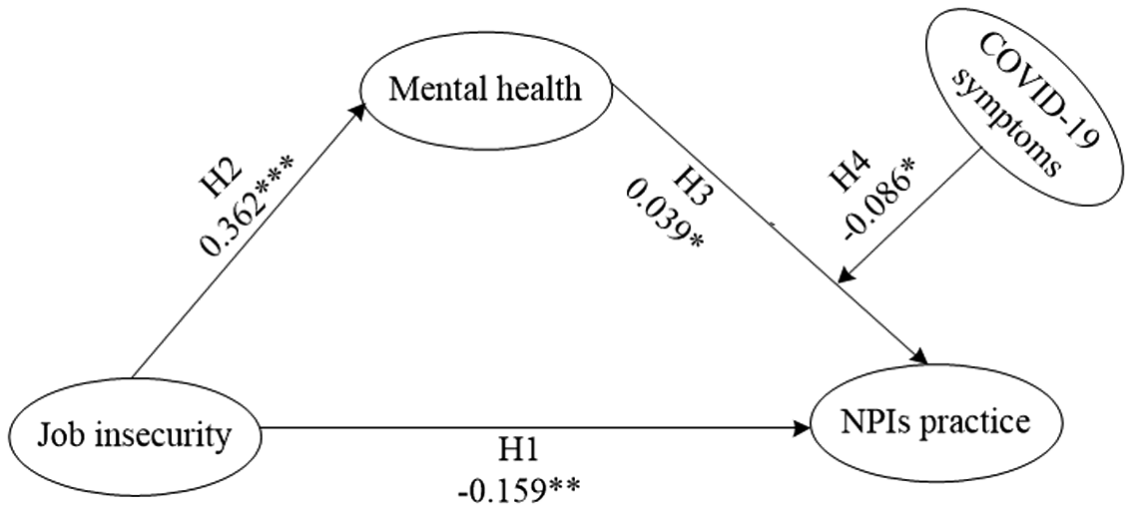
Path coefficients of hypothesized model of job insecurity, mental health, COVID-19 symptoms, and NPIs practice. ***p < 00.001, **p < 0.01, *p < 0.05.

The direct and indirect effects of job insecurity on NPIs practice along with the confidence interval estimation of the mediation and moderated mediation effects were obtained *via* 5,000 bootstrapping runs with SmartPLS. As shown in Figure 1, the direct effect of job insecurity on NPIs practice was estimated with the path coefficient β = −0.159 (*p* < 0.01), with effect size of-0.124. This finding supported the hypothesis H1 that the job insecurity was directly negatively significantly associated with NPIs practice. The effect of job insecurity on mental health was shown with path coefficient β = 0.362 (*p* < 0.001), with effect size of 0.362. This finding supported the hypothesis H2 that job insecurity was positively significantly associated with mental health. Because the hypothesis H1 was significant, mental health was found to partially mediate the relationship between job insecurity and NPIs practice with path coefficient of 0.039 (*p* < 0.045), with effect size of 0.044. This finding supported the hypothesis H3 that mental health significantly mediated the impact of job insecurity on NPIs practice.

The COVID-19 symptoms moderated mediation effect of mental health on the relationship between job insecurity and NPIs practice revealed that there was significant association with the path coefficient β = −0.086 (*p* < 0.05). The conditional effect of job insecurity on NPIs practice showed corresponding results. At the low COVID-19 symptoms level, mental health = −1, the conditional effect was-0.079 (*p* < 0.05). At the middle COVID-19 symptoms level, mental health = 0, the conditional effect was 0.040 (*p* < 0.05). At the high COVID-19 symptoms level, mental health = 1, the conditional effect was 0.081 (*p* < 0.05). These results revealed that COVID-19 symptoms as a moderator positively moderated the mediation effect of mental health over the impact of job insecurity on NPIs practice. This finding supported the hypothesis H4 that COVID-19 symptoms significantly moderated the mental health’s mediation effect on the relationship between job insecurity and NPIs practice.

## Discussions

Our results showed differences between responses of White, Black, and other minorities to factors that activate their reactions to COVID-19. It particularly highlighted that, there was racial difference in NPIs practice and job insecurity among White, Black, and other minorities. White respondents reported the highest level of NPIs practice, and the lowest level of job insecurity. These findings were in line with previous literature and public reports (Blustein et al., 2020; Couch et al., 2020). The CDC COVID-19 incidence reports showed higher rates of infection and fatality in non-White (e.g., Black, Latino, and Hispanic) than in White people (CDC, 2022). The difference between non-White and White people specifically in rates of infection, rather than severity of the illness once infected, might be associated with variations in protective behaviours practiced (Breakwell et al., 2022). Statistics showed that non-White people were more likely than White people to report difficulty to follow public health policies enacted by the government (Bhuiyan et al., 2021). The level of NPIs practice required not only protective equipment such as facemasks and disinfectant, but also the ability to stock food, water, gasoline, and medicine (Lehberger et al., 2021). To prepare those resources requires extra expenditure, which could become a sudden financial burden on lower income households. The household disposable income is lower in non-White households than their White counterparts. Accordingly, non-White people who earn low income are likely to engage in jobs such as restaurant waiter, supermarket cashier, and plumber. Therefore, the work at home orders as one of the recommended NPIs could largely affect their jobs and lead to job insecurity (Ruffolo et al., 2021). Health authorities and experts have to consider the difference in workstyle and make relevant compensation when designing protective public health policies during a pandemic.

The results of path analysis supported H1, which stated that job insecurity was negatively significantly associated with NPIs practice. This finding indicated that the higher level of COVID-19 impacts on jobs, people with lower level of job insecurity would perform more NPIs. Such relationship between higher job insecurity and lower NPIs practice, has been established in the literature (Blustein et al., 2020; Gemelas et al., 2021). During the COVID-19 pandemic, stay and work at home mandates were issued which resulted in job loss or wage reduction of people who were working in industries that are not amenable to changes of working mode (Ganson et al., 2021). People with precarious work would experience chronic stress and uncertainty, putting them at risk for violating public health policy, such as refusing to wear facemasks (Blustein et al., 2020). Our results showed that the level of job insecurity and NPIs practice were significantly different between White and Black, as well as between White and other minorities. Combining with statistical results (Couch et al., 2020), it could be that non-White had an unfavourable occupational distribution and lower level of job skills, which led to lower level of NPIs practice. Meanwhile, the public record of COVID-19 deaths confirmed that higher mortality was seen in non-White population who had barriers to perform recommended NPIs (Scannell Bryan et al., 2021). Our finding suggests that how to design a flexible public health policy on enacting containment measures for people who cannot work at home might be effective to cope with a pandemic.

The results of path analysis supported the hypothesis H2, which stated that job insecurity was positively significantly associated with mental health. This finding indicated that the higher the level of job insecurity, the worse the mental health people would have. At one point, the pandemic led to 10.1% of US workers being unemployed and faced disastrous financial burden (Carter and May, 2020). Individuals who worked to support themselves and their family members may have felt added pressure during this time, which could have increased levels of anxiety, depression, and anger (Wickens et al., 2021; Aykanian, 2022). The resolution to alleviate the association between job insecurity and mental health could be the establishment of supportive social policies. These supports for people who did not have Medicaid or unemployment insurance were confirmed to be effective to weaken depression and anxiety during the pandemic (Donnelly and Farina, 2021). In addition, people whose jobs have been either lost or downsized, not only have to worry about whether they have the means and resources to survive but also wonder whether they will catch the virus. These precarious work experience could cause chronic stress and uncertainty, putting them at risk for worsened mental health (Blustein et al., 2020). People who have reported higher level of depression, anxiety, and loneliness were inclined to engage in alcohol use and drug use, which in turn, could led to higher financial burden and unemployment rates (Tran et al., 2020). Our finding suggests that public health authorities should pay attention to the occupation distribution and employment situation to deal with mental health issues.

The results of path analysis supported the hypothesis H3, which stated that mental health mediated the impact of job insecurity on NPIs practice. This finding indicated that job insecurity is a better predictor of NPIs practice for individuals with worse mental health than for individuals with better mental health. Many studies on mental health due to COVID-19 reported the common symptom of loneliness, which was exacerbated and may be associated with other mental health outcomes (Kantor and Kantor, 2020). People who felt anxiety and loneliness were inclined to violate the recommended NPIs such as stay at home orders (Jacobson et al., 2020). Among groups who performed higher level of recommended NPIs were women, households with children, and medical staffs (Chatterjee et al., 2020; Farajzadeh et al., 2021). These groups also claimed mental health problems during the COVID-19 though, their symptoms were somewhat better than other people such as homelessness (Aykanian, 2022). As discussed above, the loss of jobs and the reduced wages could cause a sudden and huge impact on people’s financial situation and mental health (Wickens et al., 2021). People who have to worry about the inadequate resource to survive would be less likely to care about the recommended NPIs. This might also be the reason why people who lived in a poor neighbourhood tended to violate the public health policy such as self-quarantine and avoiding gathering in groups. The vicious circle between job insecurity and poor mental health would be the biggest barrier to overcome for public health authorities to control the COVID-19. Our finding suggests that maintain employment rates and income, as well as proper supportive policies on psychological health, would be the keys to enhance the practice of recommended NPIs.

The hypothesis H4 that experience of COVID-19 symptoms moderates the mental health’s mediation effect on job insecurity and NPIs practice was supported. Furthermore, our results confirmed that mental health is a better predictor of NPIs practice for individuals with a higher level of experience of COVID-19 symptoms than for individuals with a lower level of experience of COVID-19 symptoms. The finding that people with symptoms of COVID-19 experienced poorer mental health is in line with the previous literature (Moghanibashi-Mansourieh, 2020). During the early stage of COVID-19, the shortages of medical resources (e.g., staff in health care facilities, personal protective equipment), were reported all over the world (Remuzzi and Remuzzi, 2020), which could cause panic among people experiencing COVID-19 symptoms. Meanwhile, due to the lack of scientific knowledge of transmission dynamics of the virus at the time, experience of COVID-19 symptoms could provoke worry about infection (Egede et al., 2021; Sun et al., 2021). While some countries, such as China, rigorously conducted surveillance testing (Zhang et al., 2021), this was not mandated, or at least not consistent, in the US. The voluntary nature of recommended NPIs was proved to be difficult to be successful, as shown by the largest number of infected cases in the US (WHO, 2022). One in four American people have already infected with the COVID-19, and therefore, there must be more people who had experience of COVID-19 symptoms such as having friends or colleagues who infected with the virus. At the time of early September 2022, the US had fully abandoned the policy recommendation of NPIs practice. While in conversely, the population proportion of COVID-19 cases in China was quite small because public policies of NPIs practice were strictly imposed. This comparison of NPIs practice between the US and China strongly supported our results, that the more the experience of COVID-19 symptoms, the lower the level of NPIs practice. Therefore, the high risk of community transmission of the disease in the US have worsened mental health outcomes and reduced NPI practice.

## Limitation

The profile of the respondents *via* Qualtrics XM indicated there was a higher proportion of younger respondents (18–30 years), lower proportion of respondents over 51 years of age, slightly higher proportion of Whites, and much lower proportion of other minority respondents in the sample than that in the US population. Future studies might collect responses from those who are older and are minorities *via* face-to-face interviews. Another limitation was that, as a cross-sectional study, it was only able to examine associations between job insecurity, mental health and NPIs practice in the context of a certain period. The cross-sectional data have limits on accurately depicting the changing trends of people’s NPIs practice and predictive factors. A longitudinal study could be expected to reveal robust predicting factors on individuals’ NPIs practice during a pandemic. Additionally, using a survey for data collection did not allow participants to share information about their personal experiences such as community support during the pandemic and may not be able to identify the stressors they perceived with the largest impact on their mental health, and accordingly, the level of NPIs practiced. Future research could apply mixed methods such as semi-structured interviews along with the survey to collect people’s opinions on protective behavior changes during a pandemic.

## Conclusion

This study highlights the importance of investigating the impact of the COVID-19 pandemic on people’s behavior changes, as much of the literature to date has focused on cognitive factors such as risk perception, attitude, and intention to perform protective behaviors. Increasing evidence shows that the COVID-19 pandemic has caused severe economic burden, psychological depression, and adverse mental health outcomes on various groups such as college students, households with disabled children, and homelessness (Wilson et al., 2020). To our knowledge, the current study was the first to investigate the differences in job insecurity, mental health, experience of COVID-19 symptoms, and NPIs practice among White, Black, and other minorities during the pandemic in the US.

Our study has theoretical and practical implications for mitigating future pandemic risks. Regarding the theoretical implication, our cross-sectional comparisons found that, despite the indifferences in mental health, there exist significant differences in job insecurity, experience of COVID-19 symptoms, and NPIs practice among different racial groups. The results of our path analysis indicated that job insecurity was a robust predictor for protective behavior changes with NPIs practice. Such relationship was strongly impacted by the interactive effects of mental health and the experience of COVID-19 symptoms. Furthermore, it is important to understand that mental health issues were significantly complicated due to the pandemic. The public policies related to disease prevention and control (e.g., isolation) should also consider their potential effects on mental health, and could develop and adopt additional policies of social support for people who are in need.

Regarding practical implications, the risk mitigation strategies should consider adequately the negative effects on people’s mental health and physical lives. Due to the lockdowns and closures of non-essential business sectors, economic activities have been significantly negatively impacted. New business models, such as online emergency business stores during the pandemic and robust supply chain and management should be promoted. Furthermore, people’s mental health could be largely impacted by the changing environments during a pandemic. To provide community support, online mental health consultation channels should be considered. Last but not least, because the experience of symptoms of an unknown virus might cause depression and anxiety during the early stages of a pandemic, developing and deploying possible tools to ease the panic of catching the virus should also be considered. These tools should be able to disseminate scientific knowledge about the virus, and also establish online seminars to educate community leaders and the public about the new virus.

## Data availability statement

The original contributions presented in the study are included in the article/supplementary files, further inquiries can be directed to the author: wangpx@jmu.edu.

## Ethics statement

The studies involving human participants were reviewed and approved by Office of Research Integrity, James Madison University, Protocol ID: 20-1908. The patients/participants provided their written informed consent to participate in this study.

## Author contributions

YS: study design, data collection, literature review, and writing of the text. PW: study design, data collection, data analysis, and writing of the text. JT: literature review and supervise. All authors contributed to the article and approved the submitted version.

## Funding

YS acknowledges financial support from National Natural Science Foundation of China (#72204253), and Research Center for Social Development and Social Risk Control of Sichuan University (#SR21A15).

## Conflict of interest

The authors declare that the research was conducted in the absence of any commercial or financial relationships that could be construed as a potential conflict of interest.

## Publisher’s note

All claims expressed in this article are solely those of the authors and do not necessarily represent those of their affiliated organizations, or those of the publisher, the editors and the reviewers. Any product that may be evaluated in this article, or claim that may be made by its manufacturer, is not guaranteed or endorsed by the publisher.

## Footnotes

^1^ http://ssicentral.com

^2^ https://www.smartpls.com

^3^ https://jasp-stats.org/

## Appendix A

Covariance matrix of survey items.

**Table:**

	NPI1	NPI2	NPI3	NPI4	Job1	Job2	Job3	Job4	MH1	MH2	MH3	MH4
NPI1	2.861											
NPI2	1.682	2.675										
NPI3	2.035	1.807	2.998									
NPI4	1.862	1.690	2.213	2.934								
Job1	−0.552	−0.484	−0.663	−0.702	4.135							
Job2	−0.381	−0.425	−0.567	−0.521	3.363	3.685						
Job3	−0.126	−0.097	−0.305	−0.323	3.118	2.728	4.797					
Job4	−0.053	−0.021	−0.192	−0.223	2.960	2.870	3.710	4.788				
Mental health1	0.045	−0.059	−0.064	−0.072	1.082	0.951	1.162	1.024	3.041			
Mental health2	0.179	0.049	0.145	0.230	0.790	0.926	1.081	0.977	1.881	3.574		
Mental health3	−0.058	−0.188	−0.272	−0.191	1.117	1.018	1.298	1.097	1.747	1.814	3.171	
Mental health4	0.221	0.039	0.182	0.156	0.837	0.860	1.051	0.998	1.667	2.040	2.204	3.171
